# Supplementary material for: Fixation-related potentials reveal that confusing program code elicits a late frontal positivity
Source: Sci Rep. 2026 Jun 1;16:16833. doi: 10.1038/s41598-026-50946-9 (PMC13226682; doi:10.1038/s41598-026-50946-9)
Supplement: Supplementary file 1 — Supplementary Information. [file 41598_2026_50946_MOESM1_ESM.pdf]

# – Supplementary Information –

Fixation-related potentials reveal that confusing program code elicits a late frontal positivity

Annabelle Bergum, Anna-Maria Maurer, Norman Peitek, Regine Bader, Axel Mecklinger, Vera Demberg, Janet Siegmund, Sven Apel

A Stimulus Simplification and Selection

A.1 Stimulus Simplification

The code snippets in our study are based on the 41 snippet pairs from the study of Langhout and Aniche[1] on atoms of confusion in programs written in the Java programming language. To reduce both the fixations and confounding neurocognitive processes in the FRP analysis, we require stimuli with minimal size and complexity. Thus, we shortened each snippet by omitting code irrelevant to the atom of confusion (e.g., the class declaration), so that confusion arises only as a consequence of the atom and is not caused by surrounding code[2]. Furthermore, we removed print statements and named the variable of interest `R` (as in Result) to ask participants to determine its value as the output after each snippet. We further shortened the snippets to only capture the essence of each atom of confusion (e.g., replacing substring calls like `boolean V1 = "The cat is black".contains("dog")` with boolean constants `boolean V1 = false`). From the 41 simplified snippet pairs, we discarded 1 pair due to having an identical atom and a strong similarity to another pair. To ensure high reliability of our EEG data, we aimed at collecting as many data points as possible within one session. For this purpose, we created two additional variants for each snippet pair modifying several details (e.g., different integer values) without changing the atom of confusion itself, resulting in 120 snippet pairs. A pilot study showed that these modifications were sufficient to mitigate learning effects.

A.2 Stimulus Selection

To assess the suitability of the 120 simplified snippets for our main study, we performed a first pilot study with 6 participants using a Tobii EyeX eye-tracker. We presented the participants one snippet from each pair and asked them to comprehend the snippets as fast and correct as possible. During the entire study, we collected behavioral data. Furthermore, we either assessed subjective feedback after each snippet or performed a debriefing interview to gain more insight into the internal thought processes of the participant for the snippets. Then, we analyzed the obtained data to determine the suitability of the snippets. A suitable snippet must fulfill all of the following three criteria (structure of the snippets, localization of the area of interest and behavioral response), which are elaborated below.

A.2.1 Structure of the Snippets

For a high-quality ERP or FRP study, it is essential that the stimuli across conditions remain comparable to reduce the influence of confounding factors. Thus, the code structure for both conditions of a snippet pair must be comparable regarding the layout (number and length of the code lines). Therefore, among others, we excluded the conditional operators (e.g., confusing snippet using a single long statement versus clean snippet using multiple shorter statements in an if block).

A.2.2 Localization of the Area of Interest

Furthermore, to select suitable fixations for FRP analysis, a single clear area of interest must be defined in each snippet pair. For each pair, we determined areas of interest that contain either the atom of confusion (for confusing snippets) or an equivalent without the atom (for clean snippets). De Oliveira[3] showed in their eye-tracking study of atoms of confusion that the visual attention indeed concentrates in these areas. Thus, we can expect the neurocognitive processes associated with processing confusion to occur when participants fixate these areas. For two categories, this assumption does not hold: First, snippets with repeated assignments (e.g., the confusing condition containing statements without equivalent statements in the clean condition) do not possess a comparable area in the clean condition, as the atom consists of performing multiple assignments to the same variable. Second, repurposed program variables contain more than one area of interest, because the reuse of an existing variable occurs at multiple places in the code, which makes a clear decision on the location of the atom of confusion and a comparison to the clean snippet challenging. Hence, we excluded both repeated assignments and repurposed variables as atom categories for our study.

A.2.3 Behavioral Response

We tested whether the inherent assumption of confusing snippets being difficult to understand and clean snippets being easy to understand applied to our target demographics and participant sample. To this end, we excluded snippets with clear violations of the expected behavior in the pilot study, that is, snippets with less than 50% answer correctness or less than 50% easy subjective difficulty rating for either condition (e.g., due to implementation details unknown to the participants, such as variable assignment in octal system), or high complexity of the atom (e.g., storing the index for array traversal within the array itself).

We aggregated the obtained data across the snippet version to increase the statistical power. We excluded 48 of the 120 snippet pairs, because they violated one or more of our criteria, resulting in the 72 snippet pairs mentioned above. To ensure the suitability of the 72 snippet pairs and the experiment design, we conducted a second pilot study with the EEG and eye-tracking setup. In the experiment, we distributed the snippet pairs across the blocks such that each block contained only one version of a snippet pair, and the versions were counterbalanced across the participants. As piloting revealed that the learning effect was negligible for block-specific ordering, we decided to pseudo-randomly order the snippets within each experiment block independent of the other blocks.

B ERP Analysis

B.1 Results

To validate whether similar results could also be obtained without the eye-tracking alignment, we also performed an ERP analysis similar to the previous FRP analysis. As the reaction times of the participants varied, we selected a larger interval from -400 to $2,000$ ms around stimulus onset for each trial. Like in the FRP approach, we excluded trials due to artifacts in the EEG signal (66 of $1,725$) and calculated subject-wise and grand averages. As before, we performed a cluster-level paired permutation t-test, the only difference being the usage of the interval 300 to $1,500$ ms to allow the identification of longer-lasting and late onsetting ERP components. Visual inspection of the grand average wave forms suggests that the ERPs were more negative-going for confusing than for clean snippets, starting around 500 ms at frontal recording sites. However, for neither test direction, we were able to identify a significant component. The alternative hypothesis *confusing* $<$ *clean* achieved the lowest p-value ($p = 0.087 > \alpha = 0.05$) for a cluster containing mostly frontal and central electrodes in the time range 640 to 990 ms. This suggests that there are no significant amplitude differences between the confusing and the clean condition at neither recording site. Figure 1 presents the ERP waveforms at 9 electrodes and the corresponding topographic maps.

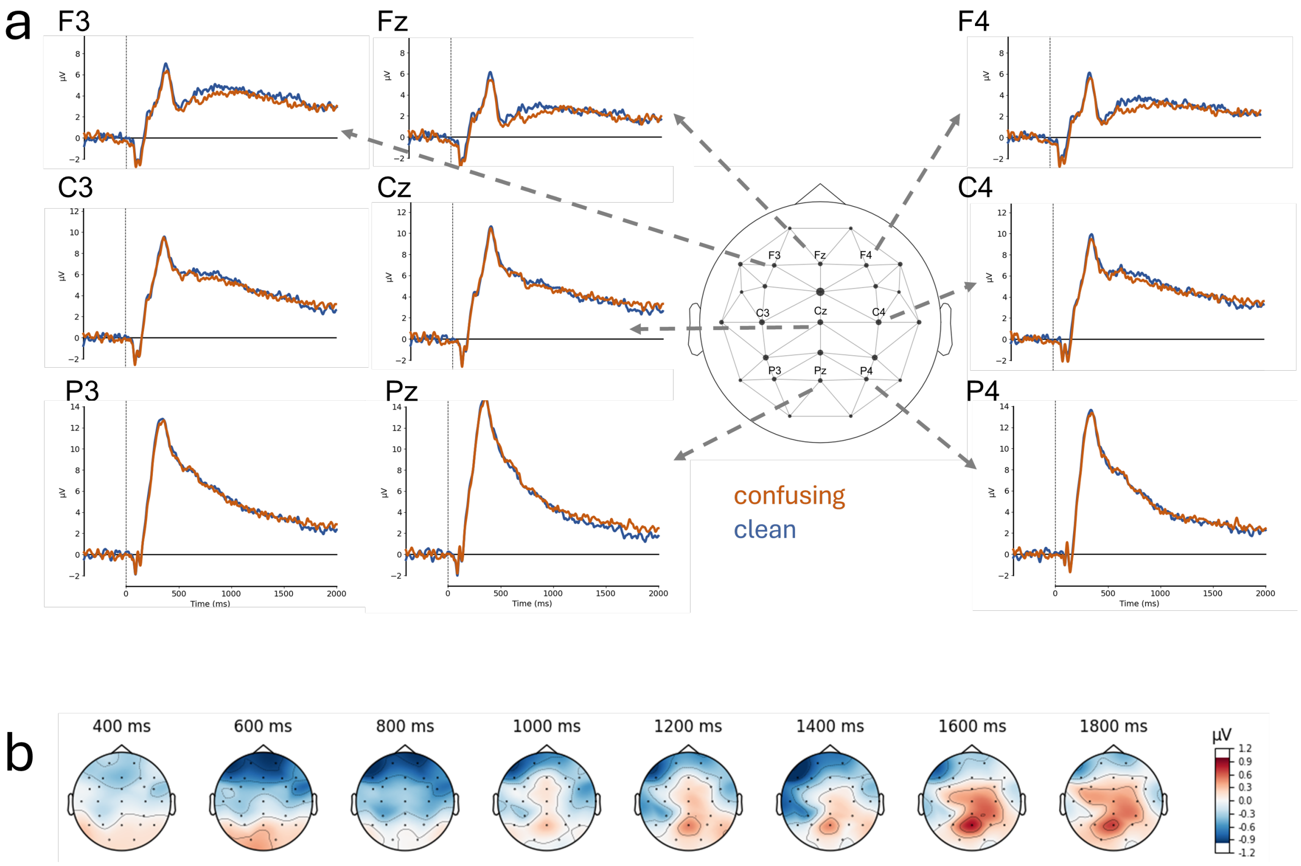

Figure 1. Part a ERP waveforms elicited by the onset of snippet presentation in a time interval from 400 ms preceding snippet onset until $1,000$ ms thereafter at 9 electrodes at frontal, central, and parietal recording sites. The ERPs are shown for confusing and clean code elements and positive polarity is plotted upwards.
Part b Topographic maps of the *confusing* $-$ *clean* difference waveform in consecutive 200 ms time intervals from 400 to $1,600$ ms after snippet onset. The onset of the interval is indicated above each map.

B.2 Methods

The procedure for ERP analysis is similar to the FRP calculation except for minor differences: Instead of using the start of a fixation, we base the epochs on stimulus onset and can thus extract epochs from $1,725$ trials of -400 to $2,000$ ms. Here, we perform no additional exclusions before creating the epochs due to missing fixations or too short intervals, as participants are required to look at each snippet for at least 3 s. After baseline correction, the epoch rejection due to artifacts excludes 66

trials, leaving $1,659$ trials for the averaging. The permutation tests were performed in the 300 to $1,500$ ms interval to detect longer-lasting components.

B.3 Differences and similarities between ERP and FRP

To put the ERP results into context, we analyzed for all $1,387$ trials included in the FRP analysis the delay between stimulus onset and the start of the first fixation in the area of interest. This delay is, in general, so large that there is no overlap between the EEG signal used for ERP compared to FRP analysis. However, this delay has a high variance within a condition $3,772\ \pm 3,367$ ms for clean and $3,246\ \pm 3,084$ ms for confusing trials across all participants (see Figure 2).

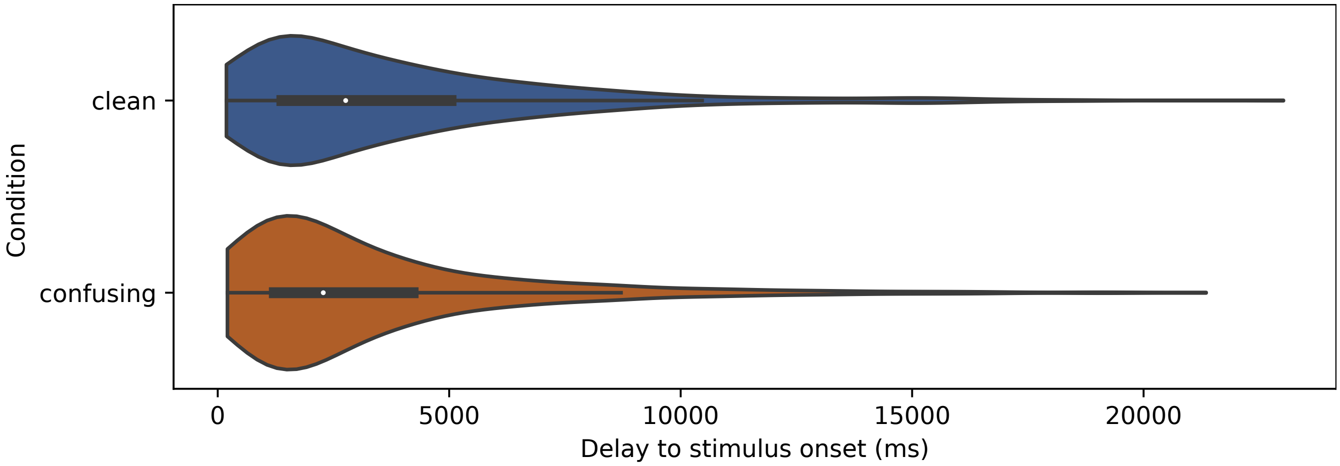

Figure 2. The delay between stimulus onset (the time-lock used for ERP analysis) and the onset of the first fixation (the time-lock used for FRP analysis) for each trial included in the FRP analysis (in ms)

C Behavioral analysis of atoms of confusion

For the behavioral analysis, we aimed at validating that the confusing snippets still carry the essence of the atom-of-confusion pattern. To this end, we captured the comprehension time, answer correctness, and the subjective difficulty rating for each trial. Confusing trials required, on average, slightly longer time to comprehend (12.2 versus 11.2 s). For answer correctness, the confusing trials showed a far lower answer correctness of 70% compared to clean trials with 88%. Participants found the confusing snippets slightly less easy to understand (82% versus 86%). For the inferential statistical analysis of the behavioral metrics, we created general linear mixed-effect regressions (GLMER) as described in Section 4.6.1 of the manuscript. The results of our final model after backward model selection for logarithmic comprehension time are presented in Table 1. We observed that confusion is present as a significant main effect ($\beta = 0.076 \pm 0.023,\ p = 0.001$), indicating that the confusing condition required a longer time to process than the clean one. Furthermore, we found a significant interaction between confusion and block number ($\beta = -0.023 \pm 0{,}010,\ p = 0.02$), which indicates that the difference between the conditions shrinks over the time-course of the experiment. Table 2 presents detailed information on random effects.

Table 1. Fixed effects of the LMER for the comprehension time model.
$ComprehensionTimeLog \sim 1 + Condition * BlockNo + BlockNo * ItemOrder + (1 + Condition \mid Participant) + (1 + Condition \mid Number)$.

	Estimate	Std. Error	t value	$Pr(>\|t\|)$	Significance
(Intercept)	1.211	0.04734	25.574	$< 2e^{-16}$	***
Condition confusing	0.07617	0.02329	3.27	0.001234	**
BlockNo	-0.1045	0.01151	-9.076	$< 2e^{-16}$	***
ItemOrder	-0.005991	0.001545	-3.879	0.000109	***
Condition confusing:BlockNo	-0.02283	0.009769	-2.337	0.019548	*
BlockNo:ItemOrder	0.001754	0.0007135	2.459	0.014052	*

Table 2. Random effects of the LMER for the comprehension time model.

Groups	Name	Variance	SD	Corr
Participant	(Intercept)	0.0150502	0.12268	
	Condition confusing	0.0007347	0.02710	0.00
Number	(Intercept)	0.0236080	0.15365	
	Condition confusing	0.0015597	0.03949	−0.28
Residual		0.0273201	0.16529	

For answer correctness, we calculated a binomial general linear mixed effects model with response accuracy as the response variable, see Table 3. We found a significant main effect of confusion ($\beta = -1.572 \pm 0.369,\ p < 0.001$), which indicates a high influence of confusion on the ability to calculate the correct output. Table 4 presents the detailed information on random effects.

Table 3. Fixed effects of the GLMER for the correctness model.
$AnswerCorrectness \sim 1 + Condition + (1 + Condition \mid Participant) + (1 + Condition \mid Number)$.

	Estimate	Std. Error	z value	$Pr(>\|z\|)$	Significance
(Intercept)	3.0374	0.4198	7.235	$4.65e^{-13}$	***
Condition confusing	−1.5716	0.3692	−4.257	$2.07e^{-05}$	***

Regarding the subjective difficulty rating, where we applied a transformation into a binary variable ($easy = 1$, $other = 0$), the final binomial model (see Table 5) contained confusion as a significant main effect ($\beta = -0.789 \pm 0.398,\ p = 0.047$), as well as trial number within a block ($\beta = -0.040 \pm 0.012,\ p < 0.001$). This indicates that participants found the confusing condition more difficult than the clean one, and they found the task increasingly easy as they progressed through the experiment. Table 6 presents detailed information on random effects.

Table 4. Random effects of the GLMER for the correctness model.

Groups	Name	Variance	SD	Corr
Participant	(Intercept)	1.0033	1.0017	
	Condition confusing	0.2941	0.5423	-0.65
Number	(Intercept)	2.2404	1.4968	
	Condition confusing	1.6341	1.2783	0.11

Table 5. Fixed effects of the GLMER for the rating model.
$SubjectiveDifficultyRating \sim 1 + Condition + ItemOrder + (1 + Condition \mid Participant) + (1 + Condition \mid Number)$.

	Estimate	Std. Error	z value	$\Pr(>\|z\|)$	Significance
(Intercept)	3.61811	0.51767	6.989	$2.76e-12$	***
Condition confusing	-0.78865	0.39765	-1.983	0.047339	*
ItemOrder	-0.04003	0.01183	-3.385	0.000713	***

Table 6. Random effects of the GLMER for the rating model.

Groups	Name	Variance	SD	Corr
Participant	(Intercept)	2.364	1.537	
	Condition confusing	1.175	1.084	-0.7
Number	(Intercept)	2.36	1.536	
	Condition confusing	1.04	1.02	-0.36

References

1. Langhout, C. & Aniche, M. *Atoms of confusion in java*. (ed.O'Conner, L.) *Proc. Int'l Conf. Program Comprehension (ICPC)*, 25–35 (2021).
2. Gopstein, D., Fayard, A.-L., Apel, S. & Cappos, J. *Thinking aloud about confusing code: A qualitative investigation of program comprehension and atoms of confusion*. (eds Devanbu, P., Cohen, M. B. & Zimmermann, T.) *Proc. Europ. Software Engineering Conf. Foundations of Software Engineering (ESEC/FSE)*, 605—-616 (Association for Computing Machinery, 2020).
3. de Oliveira, B. *et al. Atoms of confusion: The eyes do not lie*. (eds Cavalcante, E., Dantas, F. & Batista, T.) *Brazilian Symposium on Software Engineering SBES*, 243–252 (ACM, 2020).
